# Handling method alters the hedonic value of reward in laboratory mice

**DOI:** 10.1038/s41598-018-20716-3

**Published:** 2018-02-05

**Authors:** Jasmine M. Clarkson, Dominic M. Dwyer, Paul A. Flecknell, Matthew C. Leach, Candy Rowe

**Affiliations:** 1 Centre for Behaviour and Evolution, Institute of Neuroscience, Newcastle University, Newcastle upon Tyne, NE2 4HH United Kingdom; 2 School of Psychology, Cardiff University, Cardiff, CF10 3AT United Kingdom; 3 Comparative Biology Centre, Medical School, Newcastle University, Newcastle upon Tyne, NE2 4HH United Kingdom; 4 School of Natural and Environmental Sciences, Agriculture Building, Newcastle University, Newcastle upon Tyne, NE1 7RU United Kingdom

## Abstract

Mice are the most widely used model species for drug discovery and scientific research. Consequently, it is important to refine laboratory procedures and practices to ensure high standards of welfare and scientific data quality. Recent studies have identified that the standard practice of handling laboratory mice by their tails increases behaviours indicative of anxiety, which can be overcome by handling mice using a tunnel. However, despite clear negative effects on mice’s behaviour, tunnel handling has yet to be widely implemented. In this study, we provide the first evidence that tail handling also reduces mice’s responses to reward. Anhedonia is a core symptom of clinical depression, and is measured in rodents by assessing how they consume a sucrose solution: depressed mice consume less sucrose and the size of their licking bouts when drinking (their ‘lick cluster sizes’) also tend to be smaller. We found that tail handled mice showed more anhedonic responses in both measures compared to tunnel handled mice, indicative of a decreased responsiveness to reward and potentially a more depressive-like state. Our findings have significant implications for the welfare of laboratory mice as well as the design and interpretation of scientific studies, particularly those investigating or involving reward.

## Introduction

Laboratory environments can negatively impact on the behaviour, physiology, health and welfare of animals^1–4^ and considerable effort is made to regulate and improve the welfare of animals used in research laboratories around the world^5,6^. Mice are the most widely used species in biomedical research globally; in 2016 they were used in 73% of all procedures in the UK alone^7^. Consequently, understanding the experiences of mice used in research is of significant importance in order to be able to provide evidence-based improvements to housing and husbandry that will bring welfare benefits to a large number of animals, and ensure that empirical findings are robust^3,8^.

Much early work examined the housing in which mice are kept. This revealed that small cage sizes, lack of environmental enrichment, room temperatures and isolation can all negatively impact on mouse welfare, producing measurable changes in behaviour, physiology or affective state^9–12^. However, more recently, it has been proposed that the handling technique used by researchers and laboratory staff influences both the welfare of mice, and the data obtained from behavioural studies^13–16^. The standard practice of handling laboratory mice using their tails has been shown to increase anxiety compared to being handled with a tunnel or by cupping mice on the open hand^13–15^: tunnel handled mice spend more time voluntarily interacting with a handler, and show less anxiety-related behaviour in standardised behavioural tests of anxiety such as the elevated plus maze^13–15^. In addition, tail handling can reduce performance in cognitive tests, for example it has been shown to reduce the engagement of mice with a novel mouse odour in a habituation task, resulting in the impairment of the subsequent dishabituation test^15^. However, despite the evidence that tail handling can impair welfare and scientific data collection, it remains the main method used to handle mice and other more refined methods such as tunnel handling have yet to be widely implemented across research institutions.

Here, we extend previous work on handling methods to test if being handled by the tail or with a tunnel can affect the hedonic responses of mice towards a rewarding stimulus. Whilst previous studies investigating the effects of handling method on mouse welfare have measured the animals’ behaviour towards aversive experiences or punishments (such as being picked up by a handler or being placed in a novel test environment^13–15^), measuring responses to positive experiences and rewards (hedonic responses) are also important for understanding the full impact of handling methods on the affective state of an animal^17–19^. How an animal responds to both punishment and reward offers a way of accessing their enduring negative affective states: whilst anxiety and depression can both be characterized by greater expectation of punishment, depression is also associated with a reduced expectation of reward^20^. Stressful life events have been implicated in the aetiology of depression in both humans and animals^21,22^. Specifically, rodent models have shown that exposure to either a single severe (acute) stressor, or several mild (chronic) stressful experiences are sufficient in inducing a depressogenic effect^23,24^. Therefore, we predicted that a threatening and stressful stimulus, such as tail handling, could also lead to measurable changes in mouse behavior indicative of a depressed-like state.

Whilst the main approach to studying animal welfare has been to measure the presence or absence of negative affective states, recent papers have highlighted the importance of measuring welfare from their positive experiences, such as pleasure^17,18,25^. Responses towards punishing and rewarding stimuli are dissociable, and the neural pathways that deal with punishment and reward are distinct^26,27^. Therefore, the aim of this study was to test if the handling method affects the capacity of mice to experience pleasure from reward.

The reduction or inability to experience pleasure from rewarding stimuli is known as anhedonia^28,29^. Anhedonia is a core symptom of Major Depressive Disorder (MDD) in humans^29,30^, and consequently, assessing anhedonia in mice has been important for developing and validating laboratory models of depression^31^. Historically, hedonic state has been measured using voluntary consumption of sucrose solutions, under the assumption that anhedonia results in sucrose being perceived as less pleasant and consequently mice would be less motivated to drink it^22,32–34^. Animals that have undergone the chronic mild stress manipulation and show behavioural symptoms of depression, will drink less of a sweet solution than control animals^22,24,35,36^. This can be reversed through the application of anti-depressants, and has led to sucrose consumption being widely used as an indicator of affective state in rodents^32,36–40^.

Despite its widespread application and use, the sucrose consumption test is only an indirect indicator of hedonic state. This is because sucrose consumption is influenced by a number of factors: whilst the amount of sucrose solution a mouse drinks may be driven in part by how much it likes the taste, it may also be affected by motivational factors^41^ and the post-ingestive effects of the sucrose^42,43^. Therefore, researchers have sought alternative and more direct measures of hedonic responses towards tastants that are based on a more detailed examination of how an animal drinks. The orofacial movements produced upon tasting a solution and the pattern of licks during consumption are both considered to be more direct measures of palatability and hedonic responses to sucrose consumption^44–48^.

Therefore, in this study, we measured the effect of handling method not only on the amount of sucrose drunk by mice, but also on a measure of their licking behaviour considered indicative of their hedonic response to reward. When rodents drink, the pattern of licks is not random^44,46–48^. Instead they produce rhythmic sets of licks that can be grouped into clusters^44,47,48^. The number of licks in these clusters, known as ‘lick cluster size’, is positively related to the palatability of the tastant: larger lick cluster sizes are elicited by more palatable solutions^44,46–48^. Moreover, lick cluster size is also affected by the experience and physiology of the animal. This has been demonstrated through manipulations such as conditioned taste aversion which directly devalues flavours (e.g. ref.^49^) and genetic or stress manipulations thought to reduce general hedonic tone (e.g. refs^44,50^) resulting in the reduction of lick cluster sizes elicited by otherwise palatable solutions. We predicted that if handling method affects the hedonic experience of mice from drinking sucrose, then tail handled mice would have lower consumption of, and smaller lick cluster sizes towards, sucrose solutions compared to tunnel handled mice.

## Results

**Figure 1:**
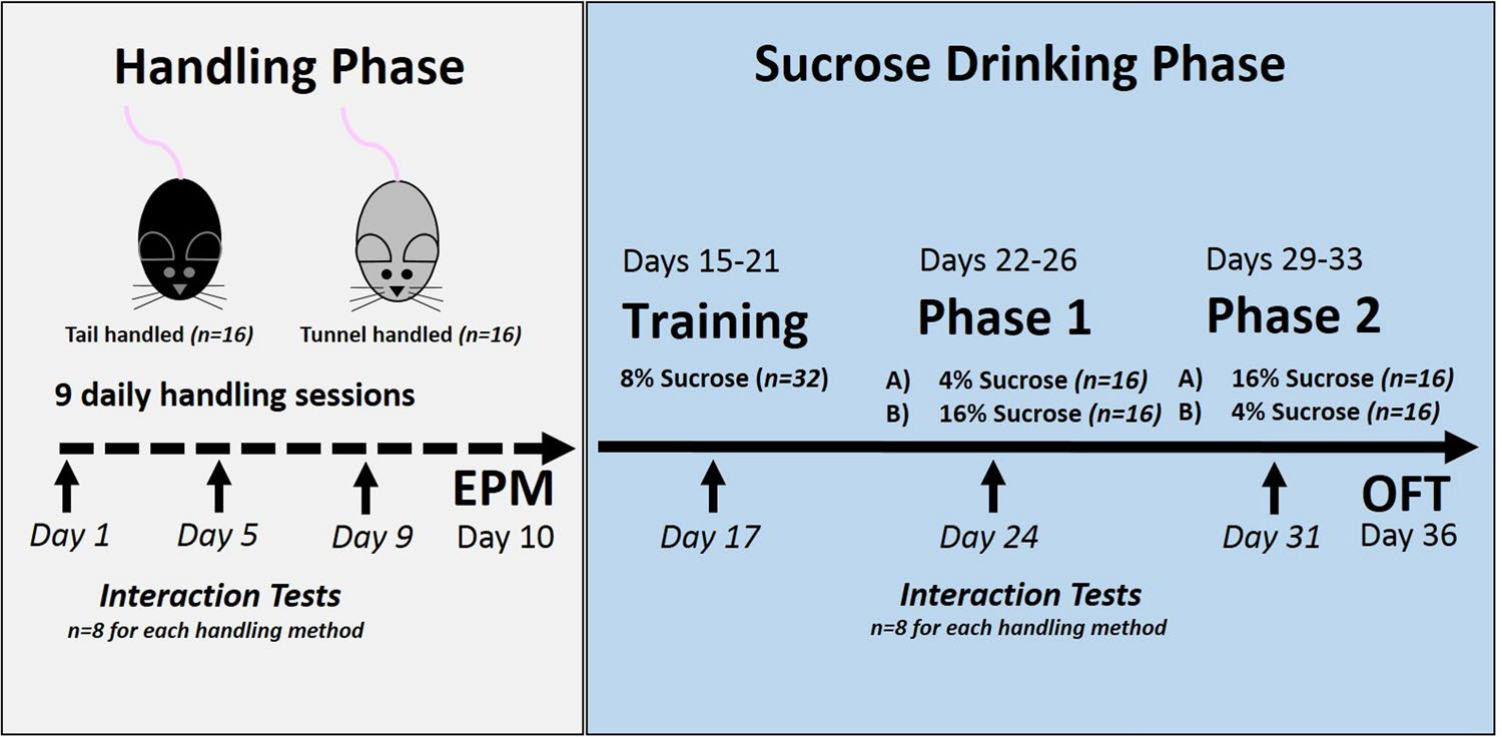
Study timeline to show the design and order of behavioural tests and sample sizes. EPM refers to Elevated Plus Maze and OFT refers to Open Field Test.

After habituation to the laboratory, mice underwent a ‘handling phase’ followed by a ‘sucrose drinking phase’ (for full details see Fig. 1). The handling phase aimed to manipulate their experiences and establish clear behavioural differences between tail and tunnel handled mice as previously reported^13,14^, before investigating anhedonic behaviour in the sucrose drinking phase.

Consistent with previous studies^13,14^, the repeated handling was sufficient to create significant differences between the two groups of mice in behavioural tests considered to be indicative of anxiety. We conducted ‘voluntary interaction tests’ on days 1, 5 and 9^13,14^, both before and after mice were handled. Tunnel handled mice spent significantly more time interacting with the handler compared to tail handled mice (ANOVA: F_1,14_ = 1062.7, p < 0.001; Figure S1; Table S1), and also showed habituation to the handler (Bonferroni adjusted pairwise comparisons day 1 versus day 5 p < 0.001; day 1 versus day 9 p < 0.001), which was absent for those handled by their tails (day 1 versus day 5 p > 0.99; day 1 versus day 9 p = 0.49) (see Supplementary Information for full details; Figure S1; Table S1). Mice were also tested in an elevated plus maze on day 10 to assess their anxiety levels. Consistent with the expectation that tail handling produces higher levels of anxiety than tunnel handling, tail handled mice showed fewer entries onto the open arms (Mann Whitney U = 174.5, p = 0.002; Figure S2A), and spent less time on them (Mann Whitney U = 175, p = 0.002; Figure S2B) (see Supplementary Information for full details; Figure S2). Taken together, these tests demonstrated that our handling manipulation was successful in eliciting differences in anxiety-like behaviours towards potentially threatening events.

**Figure 2:**
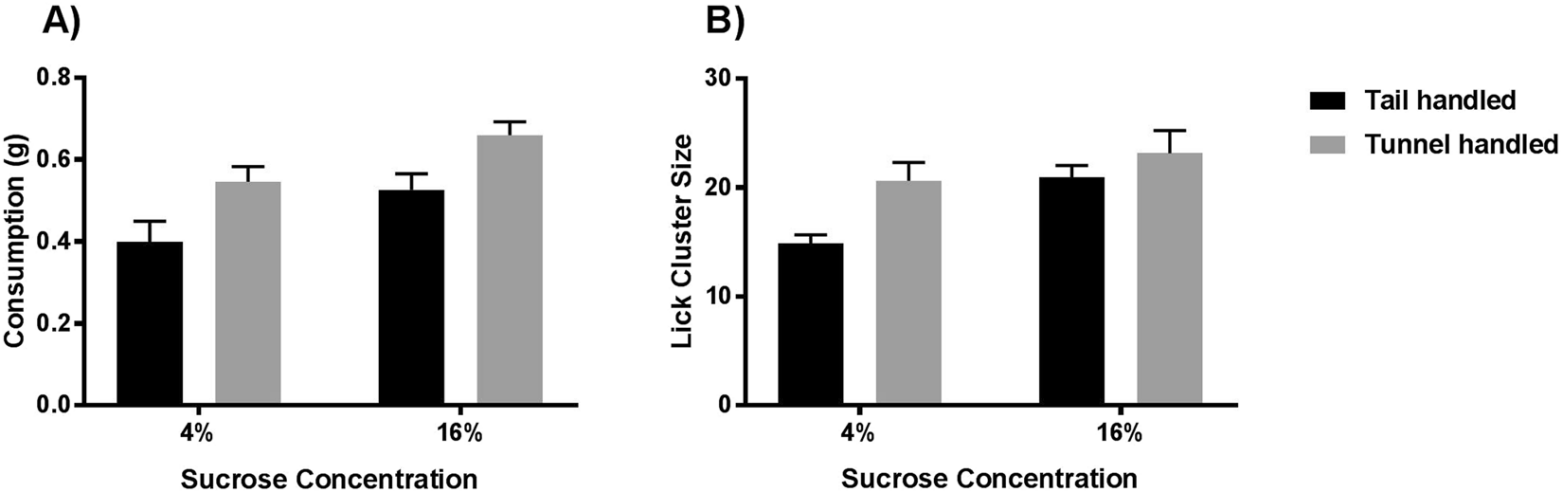
The drinking behaviour of tail and tunnel handled mice during the sucrose drinking phase. (**A**) Mean (±1 SEM) amount consumed (g). Both tail and tunnel handled mice drank more sucrose solution when given 16% compared to 4% solution (F_1,30_ = 30.817, p < 0.001), but at each concentration, tunnel handled mice drank significantly more sucrose than mice that were tail handled (F_1,30_ = 7 0.141, p = 0.012). There was no interaction between handling method and sucrose concentration (F_1,30_ = 0.100, p = 0.754). (**B**) Mean (±1 SEM) Lick Cluster Size. Both tail and tunnel handled mice had larger lick cluster sizes for 16% sucrose compared to 4% sucrose (F_1,30_ = 38.5, p < 0.001) and the tunnel handled mice had significantly larger lick cluster sizes than tail handled mice overall (250 ms: F_1,30_ = 4.6, p = 0.04). However there was a significant interaction between handling method and sucrose concentration (F_1,30_ = 10.2, p = 0.003). Tunnel handled mice only had larger lick cluster sizes than tail handled mice at the lower (4%) sucrose concentration (p = 0.003) and not at the higher concentration (p = 0.469).

Mice then entered the sucrose drinking phase, where they received daily drinking trials in custom-built test chambers. Whilst both groups of mice drank more sucrose at the higher concentration (ANOVA: F_1,30_ = 30.82, p < 0.001; Fig. 2A), tunnel handled mice drank significantly more of both sucrose solutions than mice that were tail handled (ANOVA: F_1,30_ = 7.14, p = 0.012; Fig. 2A). There was no interaction between handling method and sucrose concentration on consumption (ANOVA: F_1,30_ = 0.1, p = 0.754; Fig. 2A). These findings were qualitatively the same when controlling for the animals’ body weights (see Supplementary Information; Figure S3B), which did not significantly differ between tail and tunnel handled mice (Figure S3A).

Lick cluster sizes were also larger when mice drank the higher sucrose concentration (ANOVA: F_1,30_ = 38.50, p < 0.001; Fig. 2B) and were handled with a tunnel (ANOVA: F_1,30_ = 4.62, p = 0.04; Fig. 2B), however, there was also a significant interaction between handling method and sucrose concentration (ANOVA: F_1,30_ = 10.20, p = 0.003; Fig. 2B). This was because tunnel handled mice only had significantly larger lick cluster sizes than the tail handled mice when drinking the 4% sucrose solution (Bonferroni pairwise comparisons; p = 0.003) but not the 16% sucrose solution (p = 0.469). It is possible that we only detected the effects of handling on hedonic responses at the lower concentration because of ceiling effects at the higher concentration.

During the sucrose drinking phase, we gave mice three further voluntary interaction tests to ensure that established effects of tail and tunnel handling on measures of anxiety were still evident and had not diminished during this phase. We found that tail handled mice continued to interact significantly less with the handler compared to tunnel handled mice (ANOVA: F_1,14_ = 462.34, p < 0.001; Figure S4; Table S2), although their time spent interacting with the handler did increase over these three tests (Bonferroni adjusted pairwise comparisons for days 24 and 31 relative to day 17, p values p < 0.01) (see Supplementary Information, Figure S4; Table S2). At the end of the sucrose drinking phase, we also conducted an open field test as an independent measure of anxiety in both groups of mice. We found that tail handled mice showed significantly higher levels of anxiety, spending significantly less time in the centre of the open field compared to tunnel handled mice (Unpaired t-test: t_28_ = 3.291, p = 0.003; Figure S5B) (see Supplementary Information, Figure S5).

## Discussion

Our study provides the first evidence that handling method affects how laboratory mice perceive and respond to positive rewarding stimuli. Tail handling not only makes mice more anxious compared to tunnel handled mice^13–15^, but it also reduces their hedonic responses towards a sucrose reward. Our data show that tail handled mice drank less sucrose at both concentrations and had lower lick cluster sizes overall, although smaller cluster sizes were only evident at the lower concentration (i.e. 4%). This result could be a ceiling effect due to testing under mild water restriction, and further work would be needed in order to determine if the anhedonic effects of tail handling are moderated by the nature of the solution being consumed. However, taken together, our combined data indicate that tail handling makes mice more anhedonic and less responsive to reward compared to being handled using a tunnel. Since tail handling is the most widely used method to handle laboratory mice^51,52^, this finding has significant implications for animal welfare and the refinement of current laboratory practices, as well as scientific data collection, particularly where protocols include or investigate reward.

The presence of anhedonia in our tail handled mice, combined with increased anxiety-like behaviours relative to tunnel handled mice, is indicative of a more depressive-like state, thus suggestive of a more negative affective state in tail handled mice. This pattern of results is similar to treatments which have been explicitly designed to induce depressive-like states in rodents such as the chronic mild stress paradigm^24,35,53^ or chronic restraint^54,55^. It may therefore be surprising that tail handling could also produce results similar to these more severe experimental manipulations. However, since tail handling may mimic a predatory attack^13,56,57^, it could be that this handling method is inherently more stressful than currently thought^51^. Indeed, the degree of difference in consumption for sucrose found in mouse models of depression compared to controls, are very similar to those seen between our tunnel handled and our tail handled mice. At 4% sucrose, the reduction in tail handled mice’s consumption compared to that of tunnel handled mice was 27%. From published studies using chronic manipulations to produce models of depression in C57BL/6 mice, we have estimated the reduction in sucrose consumption relative to controls to be in the range of 33–57%^58,59^. Although these are only estimates, this does suggest that the mice could be subject to a similar depressive-like state following tail handling, whether by acute or chronic effect. This could be explored further pharmacologically by the application of anti-depressant compounds.

In addition to detecting effects of tail handling towards positive reward, we also confirmed Hurst and colleagues’ previous work and replicated their findings at a different research institution. We also found that tail handled mice interacted less with the handler and showed greater levels of anxiety in behavioural tests compared to tunnel handled mice^13–15^. However, given that our study was longer than previous published work (days 17, 24 and 31 compared to just days 1, 5 and 9), we were also able to explore the effect of tail handling on mouse behaviour over a longer period of time. The results from our open field test carried out at the end of the experiment still showed a significant effect of handling method on behavioural measures of anxiety, demonstrating that there were still clear behavioural differences. Tail handled mice continued to spend significantly less time interacting with the handler than tunnel handled mice across the entire experiment, although we did detect some changes in their behaviour during the sucrose drinking phase (see supplementary Figure S4). Tail handled mice increased the time they spent interacting with the handler over the last three voluntary interaction tests, and spent more time interacting with the handler once they had been handled. Although we are unable to draw any firm conclusions from this, there is the possibility that it may be due to forming an association with the sucrose reward, or alternatively perhaps the mice were able to learn about the sequence of events in the repeated voluntary interaction tests. Perhaps mice were more apprehensive of the handler when they were about to be picked up by their tail, compared to when they had already been picked up by their tail because they had learnt that once they had been handled, they were unlikely to be handled again.

Taken together, our data clearly demonstrate that mice are more anxious and more anhedonic when they are handled by their tail rather than by using a tunnel. This finding adds to the increasing number of studies that show that tail handling is an aversive procedure^13–16^, and that tail handling has a negative impact on the welfare of laboratory mice. It also shows that sucrose consumption and licking behaviour can be used to assess the presence of positive experiences by measuring hedonic responses towards reward. Recent papers have highlighted that to fully understand animal welfare, we need to measure positive experiences, such as pleasure^17,18,25^. The ability to assess how an animal responds to rewarding sucrose solutions offers the potential to evaluate the effects of routine laboratory conditions on their affective state and welfare.

Tail handling has been shown to affect data collection through reducing the likelihood that a mouse will engage with a cognitive task^15^. Our data suggest that the effects of tail handling may be more complex than simply not engaging with a task, but could affect how animals respond to rewards in behavioural and cognitive tasks. The vast majority of *in vivo* work involving behavioural paradigms rely on the use of reward to train the animal to perform in the given task, for example, condensed milk is often used to train mice in spatial memory tasks^60,61^ and sucrose pellets are used in operant conditioning tasks^62,63^. If tail handling reduces mice’s sensitivity for reward, this may result in longer training periods or reduced effect sizes leading to larger sample sizes being required. Tail handling may also be negatively affecting the neural circuitry underlying reward, which may mean that studies of reward pathways may not be using reliable or accurate models. We recommend that researchers consider the potential effect of tail handling on their results and interpretation of their findings.

In conclusion, we would strongly advocate that, wherever possible, mice should be handled using a tunnel and not by their tails. Tunnel handling is a simple yet effective refinement that has the potential to not only significantly improve animal welfare but also scientific data quality. Based on our own findings and those of others^13–16^, we recommend that research institutions should seek to introduce and widely implement tunnel handling as a refinement to their husbandry procedures, and that published protocols for handling mice are revised^51^.

## Methods and Materials

### Ethical Statement

Experiments were conducted at Newcastle University following approval from the universities Animal Welfare and Ethical Review Body (AWERB Project ID: 540), and in accordance with the EU Directive (2010/63/EU), ASPA (1986) and the NIH Guidelines for care and use of animals for experimental procedures. All animals were checked daily, and no adverse effects were reported. At the end of the experiment, animals were humanely killed via exposure to a rising concentration of carbon dioxide gas in accordance to Schedule 1 guidance.

### Animals, housing and husbandry

Thirty-two male C57BL/6 J mice were purchased from Charles River Laboratories, UK and were approximately 7 weeks of age (Mean ± SEM: 24.6 ± 1.6 g) on arrival. Mice were free from all recognised pathogens, and the health status of the colony was monitored following the FELASA health monitoring recommendations^64^. Mice were pair-housed in M2 cages (33 cm (L) × 15 cm (W) × 13 cm (H), North Kent Plastics), with sawdust bedding, nesting material (4HK Aspen chips, NestPak and Sizzlepet nesting, Datesand Ltd, Manchester) and a clear perspex home cage tunnel (50 mm diameter, 150 mm length) and were cleaned once per week. Animals had access to food (Special Diet Services, RM3E diet) and water *ad libitum*, except prior to drinking experiments (described below). Mice were maintained on a reverse 12:12 hour light/dark cycle (10:00 until 22:00) and experiments were conducted under red light illumination. They received relatively constant temperature (Mean ± SEM: 20.8 ± 0.7 °C) and relative humidity (Mean ± SEM: 29.3 ± 6.5%). In line with previous studies, mice were marked for identification using hair dye (Jerome Russel B. Blonde, UK) which does not interfere with the response to handling^13,14^.

### Handling Methods

After habituation to the laboratory (the animals were not handled during this time), each cage of two mice were randomly assigned (via random number generator) to one of two treatment groups, tail or tunnel handled (n = 16 per group). Mice were then only handled by their designated method (tail or tunnel handled) by the same handler wearing nitrile gloves, which were were rubbed in soiling bedding before each handling session (from mice of the same sex and strain) and a laboratory coat that was contaminated with mouse scent^13,14^. Tail handling involved grasping a mouse at the base of its tail using the thumb and forefinger, and then lifting onto the sleeve of the laboratory coat for 30 seconds before being returned to its home cage. For tunnel handling, the mouse was guided into the Perspex tunnel, and lifted above the cage and held for 30 seconds. For the first two days, the handler’s hands were loosely cupped over the ends of the tunnel to prevent escape. Mice were handled twice daily for 30 seconds, 60 seconds apart, for the first nine days. Prior to handling, the nesting material (care was taken not to disrupt the structure) and home cage tunnel were removed. This procedure was also conducted once weekly to coincide with the drinking experiments (days 17, 24 and 31). For routine husbandry practices, such as cage cleaning, mice were captured and transferred using their designated handling method either on the sleeve for tail handled mice, or in the tunnel for tunnel handled mice. The same protocol was used when transferring mice to behavioural tests, i.e. the elevated plus maze, open field test and sucrose drinking chambers.

### Voluntary interaction tests

During the handling phase (days 1, 5 and 9) and the sucrose drinking phase (days 17, 24 and 31), each cage of animals underwent ‘voluntary interaction tests’ to assess their responses to the handler. These tests allowed a comparison of behaviour in anticipation of being handled compared to after being handled on specified test days^13,14^. Each test consisted of removing the cage lid, nesting material and home cage tunnel and the handler standing motionless in front of the cage for 60 seconds. A gloved hand (tail handled) or a gloved hand holding the home cage tunnel (tunnel handled) was held resting on the substrate in the front of the cage for 60 seconds to assess voluntary interaction. Each mouse in the cage was then handled twice for 30 seconds by their designated handling method described above, before voluntary interaction was assessed again. Behaviour was filmed and later analysed using Observer XT (v11). Time spent interacting with the handler was measured for each mouse within a cage, from which an overall mean cage score was calculated. These were summed together for analyses for both tail and tunnel handled mice. Therefore, for these tests, the experimental unit was ‘cage’ (n = 8 for both groups). Interaction was defined as: sniffing (nose within 0.5 cm), touching (including paw contact), climbing on or in the handling tunnel and/or the handler’s hand. Due to the differences in how mice in the two treatments were handled during the interaction tests, the observer could not be blind to the treatment, but was blind to whether the interaction test was carried out before or after handling.

### Elevated Plus Maze

On day 10, mice underwent behavioural testing in an elevated plus maze (dimensions: arms 30 cm (L) × 5 cm (W) with side walls of 15 cm on the two closed arms, elevated 50 cm from the ground). Mice were delivered to the centre of the maze (via their designated handling method) facing an open arm and filmed for 5 mins and returned to a holding cage or the home cage, depending on whether it was the first or last mouse to undergo testing from its cage. Between subjects, the maze was cleaned with 70% ethanol and the running order was counterbalanced with respect to handling method across the testing day. Time spent in the open or closed arms was scored by a treatment blind observer using Observer XT (v11), where the time spent in an arm was defined as being when all four paws were in the arm. Three animals jumped off before the end of the test and were excluded from statistical analysis meaning the sample sizes were reduced (tail handled n = 14; tunnel handled n = 15).

### Sucrose Drinking Phase

Mice were trained and tested in eight custom made drinking chambers. These were standard mice IVC home cages (34 (L) × 19 (W) × 14 (D) cm) with clear Perspex sides, a metal perforated floor and wire cage lid with modified attachments to connect the sipper tube to the left hand side of the cage. Solutions were delivered through drinking spouts attached to 50 ml falcon tubes. Drinking chambers were connected to contact sensitive Med Associates dual contact lickometers (Med Associates Inc., St. Albans, Vermont), which transmitted the time of each lick to the nearest 0.01 second to a computer using MED-PC software. Custom-built software calculated the lick cluster sizes according to a range of interbout intervals, which is the length of time used to determine when licks can be considered to be in a single bout^44,48,65,66^. The data presented here use an interbout interval of 250 ms, meaning that any duration of 250 ms or longer between two licks defined the end of one bout and the start of the next. However, the finding were robust to the interbout interval used (see Supplementary Information; Figure S6).

Mice were separated into four groups of eight (four mice from each treatment per group) referred to as the ‘testing group’. A random number generator assigned mice into testing groups according to their cage number, meaning both mice within a cage were assigned to the same testing group and were tested in the same order and time each day. Water bottles on the home cage were removed 2 hours prior to sucrose drinking trials, before the lights went off to encourage consumption. Mice were trained across seven consecutive days for 15 minutes each day (Days 15–21) to drink sucrose (8% (w/w) sucrose solution) from the spouts. During the first three training sessions the spout was left to protrude into the cage to ensure engagement with the task, after this the spout was flush with the cage lid in order to reduce accidental contact. Once all animals were engaged with the task and consistently drinking (>100 licks), the sucrose drinking phase began.

Sucrose drinking testing had two phases, where mice were tested on all 5 days for each phase. Phase 1 (Days 22–26) consisted of half the animals (n = 16; 8 from each handling method) receiving 4% (w/w) sucrose and half (n = 16; 8 from each handling method) receiving 16% (w/w) sucrose for 15 minutes. This was balanced with regards to treatment group and across testing groups. Phase 2 (Days 29–33) reversed the sucrose concentration. We used two concentrations of sucrose to assess the responses to stimuli with differing hedonic properties. We measured the mass of sucrose solution consumed and the timing of each lick in every test trial; from this, we calculated the mean consumption of sucrose (g) and the mean lick cluster sizes for each animal across the five days at both concentrations for use in our analyses.

### Open Field Test

On day 36 each mouse was individually placed via their designated handling method in the centre of a rectangular arena, (54.5 cm (L) ×  35.5 cm (W) × 17 cm (H)) made of white plastic with a transparent Perspex lid for 10 minutes. The order was counterbalanced with respect to handling method. Behaviour was filmed and analysed using Ethovision XT (v 5.1) which automatically tracked the time spent in the centre, relative to the periphery. Presence of defecation during the open field test was noted and later analysed. Due to a technical error, videos were only scored for 14 out of the 16 tail handled mice.

### Statistical Analyses

All statistical analyses were conducted using IBM Corp. SPSS (v23, SPSS Inc, Chicago, USA). Datasets were tested for normality and homogeneity of variance, where assumptions were not met data were transformed or non-parametric statistical methods used. Where significant main effects were found, Bonferroni post hoc tests were performed to look at pairwise comparisons between variables. Refer to Table 1 for full statistical analyses.

**Table 1:** Statistical tests for each data set with respective factors, experimental unit and sample size.

Data	Dependent Variable	Statistical Test	Factors	Experimental Unit	Sample Size
Voluntary interaction tests	Percentage time spent interacting	Repeated measures ANOVA	Between subject factors: Handling method (2 levels) Within-subject factors: day (6 levels), time (2 levels: pre or post handling)	Cage	n = 8 tail handled n = 8 tunnel handled
Elevated plus maze	Number of open arm entries; Duration on open arms	Mann-Whitney U test	Handling method (2 levels)	Mouse	n = 14 tail handled n = 15 tunnel handled
	Number of protected stretch attend postures	Unpaired t-test	Handling method (2 levels)	Mouse	n = 14 tail handled n = 15 tunnel handled
	Number of mice that defecated	Binary logistic regression	Handling method (2 levels)	Mouse	n = 14 tail handled n = 15 tunnel handled
Sucrose drinking	Consumption (g); Lick cluster size (log transformed)	Repeated measures ANOVA	Between subject Factors: Handling method (2 levels) Within-subject Factors: Sucrose concentration (2 levels)	Mouse	n = 16 tail handled n = 16 tunnel handled
Open field test	Duration of movement; Duration in centre; Crosses to centre; Distance travelled; Mean velocity	Unpaired t tests	Handling method (2 levels)	Mouse	n = 14 tail handled n = 16 tunnel handled
	Number of mice that defecated	Binary logistic regression	Handling method (2 levels)	Mouse	n = 16 tail handled n = 16 tunnel handled

### Data availability

All data generated or analysed during this study are freely available on the Zenodo repository: 10.5281/zenodo.1157907.

## Electronic supplementary material

Supplementary Information
