# Supplementary material for: Handling method alters the hedonic value of reward in laboratory mice
Source: Sci Rep. 2018 Feb 5;8:2448. doi: 10.1038/s41598-018-20716-3 (PMC5799408; doi:10.1038/s41598-018-20716-3)
Supplement: Supplementary file 1 — Supplementary Information [file 41598_2018_20716_MOESM1_ESM.pdf]

Supplementary Information

“Handling method alters the hedonic value of reward in laboratory mice”

Authors: Jasmine M Clarkson, Dominic M Dwyer, Paul A Flecknell, Matthew C Leach, Candy Rowe

Changes in measures of anxiety arising from the handling phase

We established that the mice were influenced by the handling method prior to the start of the sucrose consumption trials through two different behavioural tests.

In the 'voluntary interaction tests' conducted on days 1, 5 and 9, we found that tunnel handled mice spent significantly more time voluntarily interacting with the handler than tail handled mice (Table S1; Figure S1). This difference was evident even on the first day, which was in line with previous findings [1,2]. This difference is likely due to the familiarity of the tunnel in the home cages [2]. Time spent interacting changed across days (Table S1; Figure S1), but a significant interaction between day and handling method indicated that the degree of change across days differentially affect the tail and tunnel handled mice (Table S1; Figure S1). Whilst the tunnel handled mice spent significantly more time interacting with the handler on all subsequent days following day 1 (Bonferroni adjusted pairwise comparisons day 1 versus day 5 $p<0.001$; day 1 versus day 9 $p<0.001$), tail handled mice did not (day 1 versus day 5 $p>0.99$; day 1 versus day 9 $p=0.49$). We found no other significant main effects or interactions (see Table S1).

Table S1: The statistical reports for the analysis of the interaction tests for days 1,5 and 9

Factor	F_{df}	p value
Handling method	$F_{1,14} = 1062.7$	<0.001 ***
Day	$F_{1,28} = 29.03$	<0.001 ***
Time (pre or post handling)	$F_{1,14} = 0.60$	=0.45 ns
Handling method x Day	$F_{1,28} = 23.67$	<0.001 ***
Handling method x Time	$F_{1,14} = 0.04$	=0.85 ns
Day x Time	$F_{1,28} = 1.30$	=0.29 ns
Handling method x Day x Time	$F_{1,28} = 0.11$	=0.89 ns

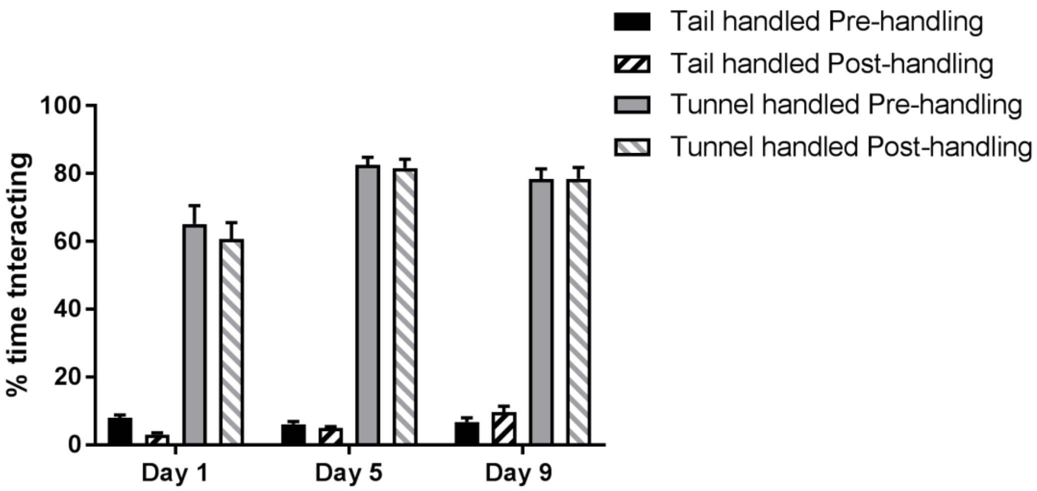

Figure S1. Mean (±1SEM) time spent interacting with the handler in the voluntary interaction tests conducted on three different days in the handling phase. Interaction tests were conducted both before (pre-) and after (post-) the animals were handled via the tail or tunnel handling method.

On day 10, mice were individually placed in an elevated plus maze. In line with previous findings[1,2], compared to tail handled mice, tunnel handled mice had a significantly greater number of entries onto the open arms (Mann Whitney U = 174.5, p = 0.002; Figure S2A) and spent longer on those arms (Mann Whitney U = 175, p = 0.002; Figure S2B). However, although in the predicted direction, the number of protected stretch attend postures ($t_{27} = 1.718$, $p = 0.097$; Figure S2C) and defecation events (Figure S2D) did not significantly differ between our two handling methods.

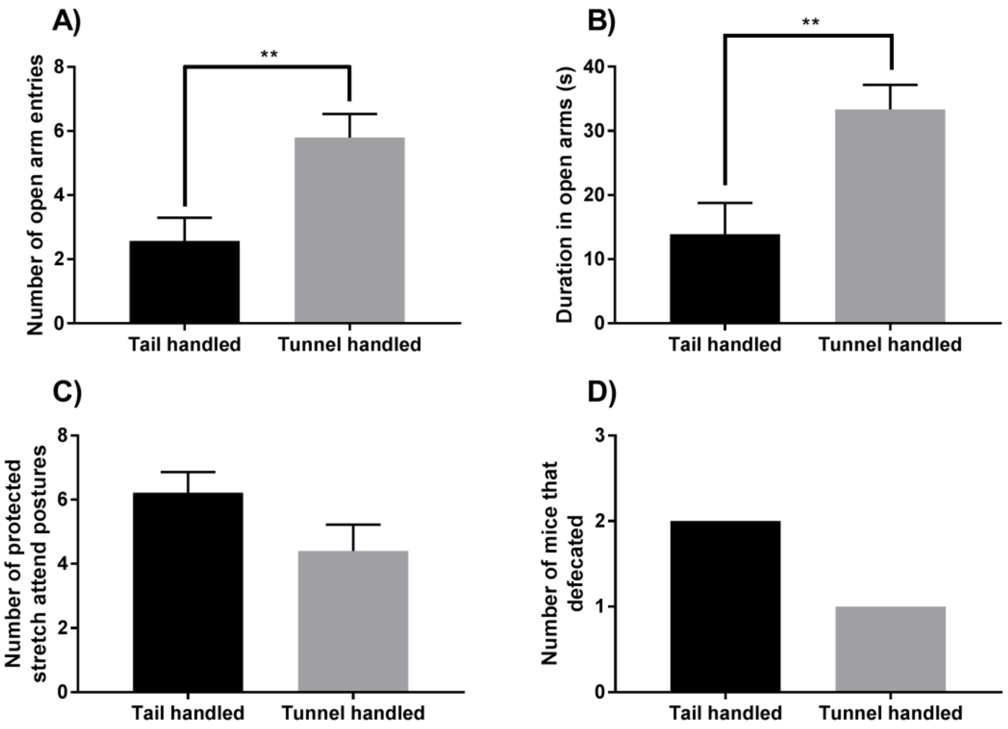

Figure S2. Results from tail handled and tunnel handled mice placed in the elevated plus maze on day 10. **A.** Mean (±1SEM) number of entries into the open arms. **B.** Mean (±1SEM) length of time spent in the open arms. **C.** Mean (±1SEM) number of stretch attend postures. **D**. Total number of mice that defecated during testing.

Body weight had no effect on total sucrose consumption

We found that handling method had no significant effect on the mean body weight of the animals, although an independent samples t-test revealed there was a tendency for tail handled mice to be heavier than tunnel handled mice ($t_{30} = 1.905$, $p=0.066$; Figure S3A). To be sure that bodyweight was not influencing our sucrose consumption data (e.g. by larger mice requiring more calories and being more motivated to drink), we also analysed these data when controlling for body weight. The data was analysed using a two way ANOVA with sucrose concentration as the within subject factor and handling method as the between subject factor. The results were qualitatively the same as those presented for total consumption of the sucrose solutions in the main manuscript (Figure 1A). Both tail and tunnel handled mice drank significantly more of the higher sucrose concentration ($F_{1,30} = 32.56$, $p<0.001$; Figure S3B), but tunnel handled mice drank significantly more sucrose irrespective of concentration compared to tail handled mice ($F_{1,30} = 13.43$, $p=0.001$; Figure S3B). Again, we found no interaction between handling method and sucrose concentration ($F_{1,30} = 0.02$, $p=0.887$; Figure S3B).

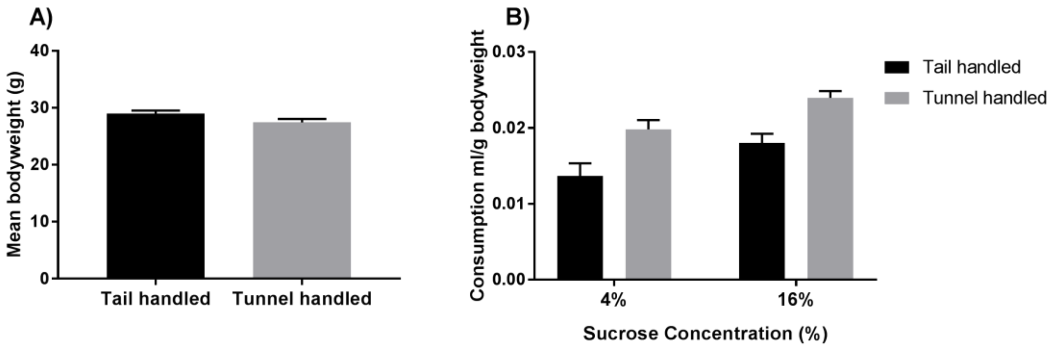

Figure S3. A. Mean (±1SEM) consumption of sucrose solutions during the sucrose drinking phase normalised for body weight (ml/g). **B.** Mean (±1SEM) body weight (g) for tail and tunnel handled mice during sucrose consumption phase. The mean body weight was derived from the body weights taken during the two phases of the sucrose consumption phase.

Measures of anxiety during and immediately after the sucrose consumption phase

We used voluntary interaction tests during the sucrose consumption phase and an open field test immediately after to ensure that mice showed continued differences in behavioural tests associated with anxiety.

In the voluntary interaction tests conducted on days 17, 24 and 31, again tunnel handled mice continued to spend significantly more time voluntarily interacting with the handler than mice handled via their tails (Table S2; Figure S4). We also found that the amount of time spent interacting depended on the time of the test, i.e. whether it was before or after handling (Table S2), however this differed according to the way mice were handled (Table S2). Pairwise comparisons revealed

that the amount of time spent interacting was different between pre and post handling for the tail handled mice (Bonferroni adjusted p values p=0.002) only (tunnel handled mice p=0.691). Furthermore, although we found no significant effect day on the amount of voluntary interaction overall (Table S2) this did interact with handling method (Table S2). Pairwise comparisons revealed that, irrespective of the time of the test, tail handled mice spent significantly more time interacting with the handler on days 24 and 31 relative to day 17 (Bonferroni adjusted p values p<0.01) yet tunnel handled mice spent significantly less time interacting with the handler on day 31 relative to day 17 (p=0.043). We found no other significant main effects or interactions (see Table S2).

Table S2: The statistical reports for the analysis of the interaction tests for days 17, 24 and 31

**Factor**	**F_{df}**	**p value**
Handling method	$F_{1,14} = 462.34$	<0.001 ***
Day	$F_{1,28} = 1.36$	=0.27 ns
Time (pre or post handling)	$F_{1,14} = 5.4$	=0.036 *
Handling method x Day	$F_{1,28} = 19.73$	<0.001 ***
Handling method x Time	$F_{1,14} = 8.39$	=0.012 *
Day x Time	$F_{1,28} = 3.06$	=0.063 ns
Handling method x Day x Time	$F_{1,28} = 1.40$	=0.26 ns

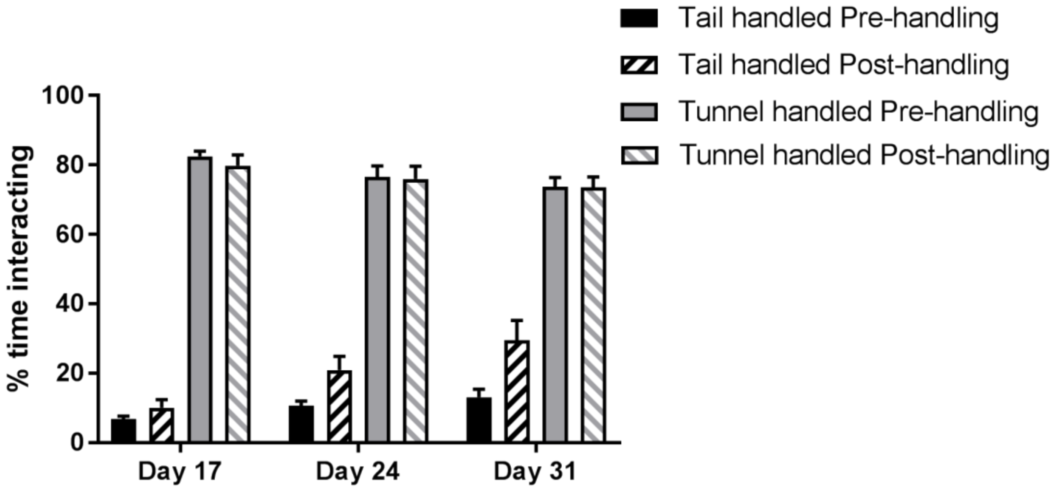

Figure S4. Mean (±1SEM) time spent interacting with the handler in the voluntary interaction tests conducted on three different days during the sucrose drinking phase. Interaction tests were conducted both before (pre-) and after (post-) the animals were handled via either the tail or tunnel handling method.

In the open field test, although the time that mice spent moving did not significantly differ between the handing methods (t_{28}=0.860, p=397, Figure S5A), their patterns of movement were very different. Tunnel handled mice spent significantly longer in the centre of the arena (t_{28}=3.291, p=0.003; Figure S5B) and performed significantly more crosses into the centre (t_{28}=5.095, p<0.001; Figure S5C). They also travelled significantly further (t_{28}=4.361, p<0.001; Figure S5D) and had a significantly higher mean velocity when travelling (t_{28}=4.526, p<0.001; Figure S5E) than tail handled mice. In all of these tests, tail handled mice showed a tendency to defecate more, which is also an indication of stress [3] (Figure S5F). Taken together, tail handled mice showed more anxiety-like responses and greater thigmotaxis in the open field compared to mice handled by a tunnel.

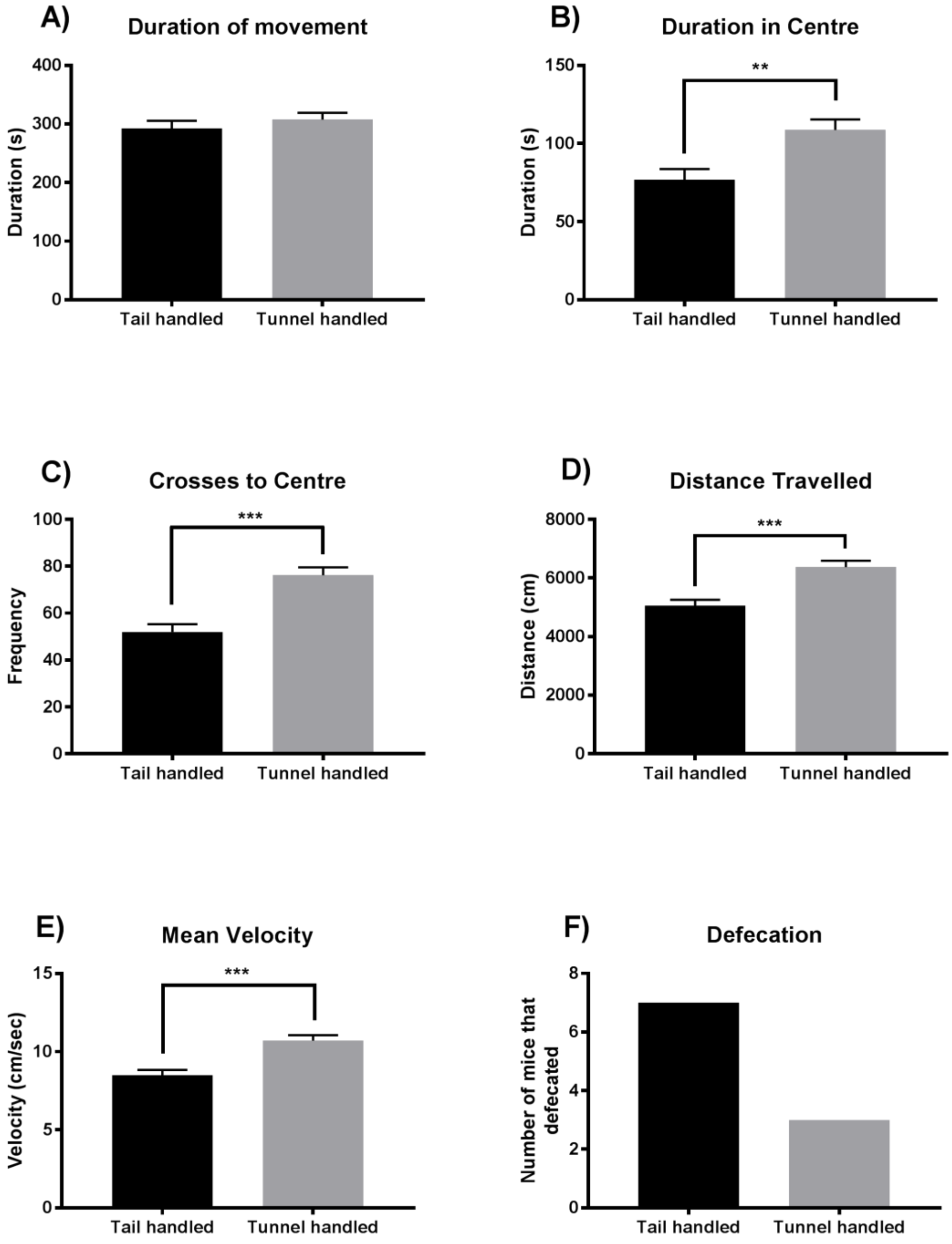

Figure S5. The behaviour of tail and tunnel handled mice in the open field test. **A.** Mean (±1SEM) length of time spent moving. **B.** Mean (±1SEM) length of time spent in the centre. **C.** Mean (±1SEM) number of crosses to the centre. **D.** Mean (±1SEM) distance travelled. **E.** Mean (±1SEM) velocity when moving. **F.** Total number of mice that defecated during testing.

Use of different inter-bout intervals did not change the interpretation of lick cluster size results

There are a number of different inter-bout intervals that can be used to determine a single bout of licking, and thus determine lick cluster size. Therefore, we checked to see that the results presented in the main manuscript were not specific to the inter-bout interval used (250ms). Irrespective of the criterion used to define a single bout of licking, i.e. up to or larger than 250ms (Figure S6A), 500ms (Figure S6B) or 1000ms (Figure S6C), interpretation of the results remained the same (Table S3).

Table S3. The statistical reports for each inter-bout interval criterion for lick cluster size

Inter-bout interval	Factor	F_{df}	p value
250ms	Handling method	$F_{1,30} = 4.62$	0.04 *
	Sucrose concentration	$F_{1,30} = 38.5$	<0.001 ***
	Handling method x sucrose concentration	$F_{1,30} = 10.2$	0.003**
500ms	Handling method	$F_{1,30} = 4.16$	0.05 *
	Sucrose concentration	$F_{1,30} = 70.11$	<0.001 ***
	Handling method x sucrose concentration	$F_{1,30} = 11.44$	0.002**
1000ms	Handling method	$F_{1,30} = 5.78$	0.023 *
	Sucrose concentration	$F_{1,30} = 60.25$	<0.001 ***
	Handling method x sucrose concentration	$F_{1,30} = 10.2$	0.003 **

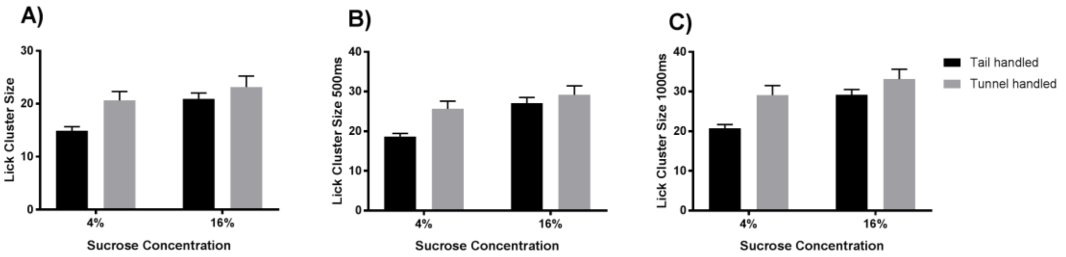

Figure S6. Mean (±1SEM) Lick Cluster Size for tail and tunnel handled mice for both sucrose concentrations according to three different inter-bout interval criteria. Whereby a bout of licking is classified as being at least **A.** 250ms **B.** 500ms or **C.** 1000ms in length, to determine mean lick cluster size.

References

1. Hurst, J. L. & West, R. S. Taming anxiety in laboratory mice. *Nature* (2010). doi:10.1038/NMETH.1500
2. Gouveia, K. *et al.* Reducing Mouse Anxiety during Handling: Effect of Experience with Handling Tunnels. *PLoS One* **8,** e66401 (2013).
3. Henderson, N. D., Turri, M. G., Defries, J. C. & Flint, J. QTL Analysis of Multiple Behavioral Measures of Anxiety in Mice. *Behav. Genet.* **34,** (2004).
